# Patient's Knowledge and Attitude towards Tuberculosis in an Urban Setting

**DOI:** 10.1155/2012/352850

**Published:** 2012-12-31

**Authors:** Saria Tasnim, Aminur Rahman, F. M. Anamul Hoque

**Affiliations:** ^1^Department of Obstetrics & Gynaecology, Institute of Child and Mother Health, Matuail, Dhaka 1362, Bangladesh; ^2^Centre for Injury Prevention Bangladesh, Bangladesh

## Abstract

Tuberculosis is a public health problem in Bangladesh. This cross-sectional study was conducted to assess knowledge of TB patients about symptoms, ways of transmission and treatment of tuberculosis, and their perception of the illness. Between March and August 2008, 762 adult TB patients were interviewed at selected DOTS centre of Dhaka city. Male and female distribution was 55.6% and 44.4%, respectively. One quarter of them were illiterate, and more than half had extended family and live in a congested situation. Night fever was the most common symptom known (89.9%), and 56% were aware that it could spread through sneezing/coughing. Television was mentioned as a source of information about TB. The majority expressed a helping attitude towards other TB patients. Although most of them were positive about getting family support, 46.6% mentioned discrimination of separate utensils for food or drink. About 50.5% expressed increased sadness, 39.8% had fear of loss of job/wedges, and 21.4% felt socially neglected. Along with drug treatment the psychosocial reactions of TB patients should be addressed at DOTS centers for better control of the disease.

## 1. Introduction 

Tuberculosis (TB) is a public health problem in many developing countries including Bangladesh. Globally there were 8.8 million incident cases of TB in 2010 [1]. With the rising number of HIV infection and AIDS cases there is a threat of resurgence of TB as this is the most common opportunistic infection in them [2]. TB is the leading cause of death among all infectious diseases and WHO reported that in 2010 there were 1.1 million deaths among HIV-negative people and an additional 0.35 million deaths from HIV associated tuberculosis [1].

The global burden of TB mainly lies in the 22 high burden countries and about 50% of prevalence occurs in 5 countries of South East Asia, namely, India, Indonesia, Bangladesh, and Thailand, Myanmar. Bangladesh rank sixth among the high burden countries with an incident rate of 225 per 100,000 thousand population per year and a mortality rate (exclusive of HIV) of 43 per 100,000 thousand population per year [1].

Millennium development goal 6 implies to halt and begin to reverse the incidence of TB by 2015 and fixed the target (MDG 6 Target 6.C) to reduce prevalence of and death due to TB by 50% compared with a baseline of 1990 by 2015 [3]. The direct observed treatment short course (DOTS) was launched in 1995 as the main strategy in the control of tuberculosis [4]. The strategy includes diagnosis through bacteriology and standardized short-course chemotherapy with full patient support [4].

Bangladesh adopted DOTS strategy in national TB control program (NTP) during fourth population & health plan (1992–1998) and integrated into essential service package under the health and populations sector program (HPSP) in 1998 [5]. Although initially TB services were based in TB clinics and TB hospitals, under the DOTS strategy the services were expanded gradually to primary level of health facility incorporating GO-NGO partnership. Government and NGO community health workers are involved in village level for case detection and awareness building activities. In 2002, DOTS was expanded to Dhaka metropolitan city. By 2006 entire country has been covered by DOTS service [5]. 

The DOTS strategy relies greatly on passive case finding for TB treatment and its success depends on the patient's health awareness, ability to recognize early sign symptoms, and accessibility to health services for immediate self-reporting [6]. It is important that basic knowledge about the disease and the availability of treatment is clear among community to prevent any undue delay in availing the service. The perceptions of TB prevailing in the community influence the health seeking behavior of people for their symptoms. While care seeking behavior of chest symptomatic has been explored in different studies, there is dearth of information on community perceptions of TB [7]. The current study was done to determine knowledge of TB patients about tuberculosis and their perception of the illness.

## 2. Method

 This was a cross-sectional descriptive study conducted during March to August 2008 in selected DOTS centres of Dhaka metropolitan city. From the list of 73 centres providing DOTS service 27 were selected according to convenience and accessibility. Face to face interview of adult TB patients attending the selected centre for treatment was taken using structured questionnaire. Written informed consent was obtained from all respondents. Data was analysed using SPSS software version 12.

## 3. Results

**Figure 1:**
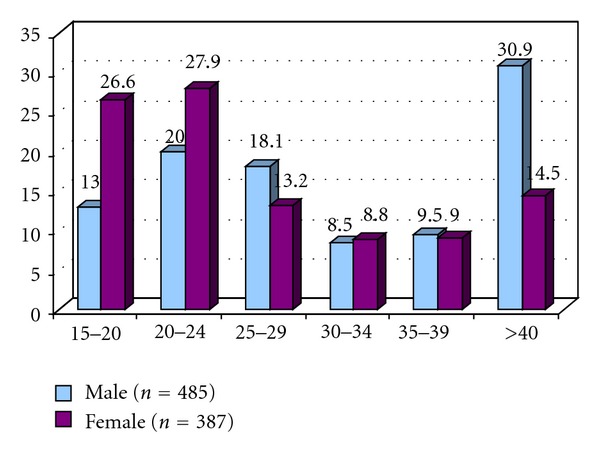
Age distribution (*N* = 872).

**Figure 2:**
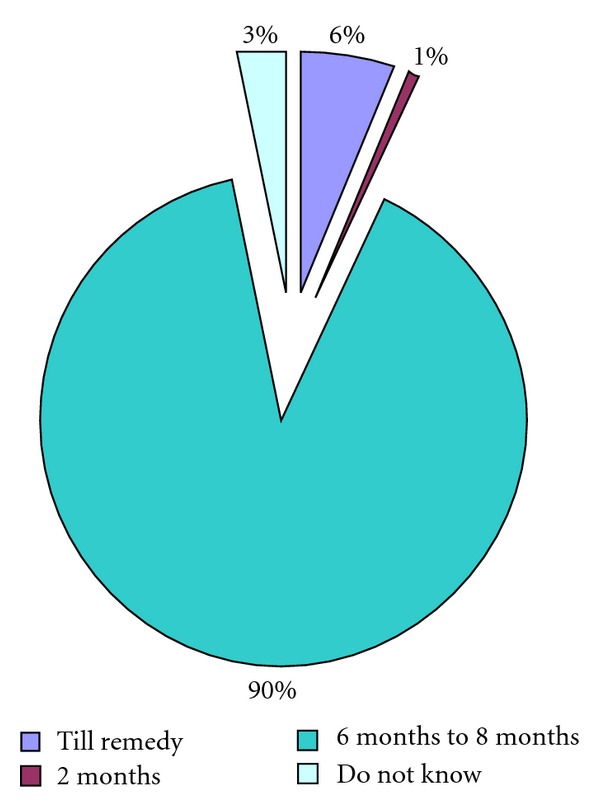
Knowledge about duration of TB treatment (*N* = 872).

Total number of respondents were 872 constituting 55.6% male and 44.4% female, respectively, and more than half of them (58 > 4%) were within 15–29 years (Figure 1). One fourth of them were illiterate, about 70% studied in any institution, mean family size was 4.89 ± 1.8 and 5.03 ± 1.9 among male and female, respectively (Table 1). About 46.8% stated that they get information about TB from television, next was doctors chamber (18.2%), and 87% mentioned about bill boards (Table 2). Regarding symptoms of TB (89.9%) mentioned night fever, tiredness (86.5%), productive cough (80.6%), and (61.6%) mentioned cough more than 3 weeks (Table 3). About mode of transmission of disease 22.9% were ignorant, 56% thought sneezing and cough, smoking 5.4%, and 2.2% mentioned TB is a familial disease (Table 4). Most of them knew that TB can be cured completely, they opined that the remedial measure is taking specific drugs given in DOTS centre (Table 5). Ninety percent of them can mention the duration of treatment should be 6–8 months (Figure 2). Regarding attitude towards other TB patients 65.7% felt compassionate and desire to help, 28.6% indifferent, and 4.9% would prefer to stay away (Table 6). About self-perception of being TB patient 95.4% got family support, 59.3% are anxious for reduction of family income, 21.9% felt socially neglected, 46.6% expressed that utensils for food/drink are separated for them, and 11.2% felt isolated within family (Table 7).

## 4. Discussion

Tuberculosis (TB) especially affects the economically most productive age group. The Bangladesh national tuberculosis program has reported that among TB cases three fourth belonged to age group 15–45 years [5]. In the current study, the mean age of the patients was 30.65 ± 13.1 years ranging from 15 to 86 years and female patients were younger than the male patients (*P* < 0.05). Other study from Bangladesh reported 70% cases were within age group 15–44 years and mean age was 36 years [8]. Karim et al. reported mean age for men and women was 41.8 and 33.6 years and among women more teen-agers were diagnosed [6]. Study from India showed a mean age of 43.02 years (range: 20–90 years) [9]. A study from Nigeria reported mean age of male and female 33.5 and 22.2 years, respectively [2].

Studies show prolonged cough, at times chest pain, loss of weight, fever, difficulty in breathing, and coughing up blood are perceived to be associated with TB by the people [7, 10, 11]. In the present study the symptoms of TB reported by the patients indicated a fairly good level of knowledge. This may be associated with urban setting of the study with better opportunity to access to information and education level of respondents. Croft reported 44% individuals to be aware of cough as TB symptom in a rural area of Bangladesh [12]. Study from India reported that 73.7% cough with sputum, weakness and breathlessness 40.4%, fever 34.3%, and haemoptysis 30% were mentioned as symptoms of TB [9]. In Pakistan most commonly recognized symptom was cough 83.5%, fever 54.7%, chest pain 24.7%, and bloody sputum 24.7% [11].

 However, misconceptions about the cause and mode of transmission are also prevalent. In some places TB is believed to be hereditary [10, 13, 14]. Some studies found cause of TB was attributed to smoking and drinking alcohol [7, 15] stamping on sputum [7], sharing eating and drinking utensil, and sleeping with TB patient [11, 16]. Study from Vietnam brought out that men have wider social contacts as compared to women and were more likely to get TB than women [17]. Poor knowledge about TB and traditional misbelieves are associated with delays in case detection [10, 18].

Mass media could play a vital role in success for passive case finding and treatment [3]. In our study television was cited as the main sources of information (46.8) and a small proportion mentioned about radio and bill boards. This reflects positive impact of governments' initiatives of mass awareness utilizing the media. This may also be the reason that 98% could mention that TB can be cured completely through taking specific drugs from DOT centers. In India doctors and health care workers were stated to be the source of the information regarding tuberculosis by 50.2% followed by mass media (33.8%), and (34.7%) mentioned interaction with others in the community [9].

Tuberculosis-related pervasive stigma may worsen the quality of life of its victims [15]. A higher degree of psychiatric morbidity like denial, hopelessness about life, tension/anxiety, and feeling neglected by family and society is common in TB patients [19]. Eram et al. reported the initial reaction to the diagnosis was negative in majority of patients, 98% were hopeful of care, 30% had anxiety/tension, 26% had lost interest from life, and 20% could not explain how they felt [20].

Being diagnosed with TB can create the fear of isolation and discrimination [2]. In HIV prevalent countries TB patients are stigmatized due to assumed coinfection with HIV [10]. Study from Uganda shows the main reason for delayed diagnosis of TB was a lack of recognition of symptoms and the stigma of association with HIV [21]. We do not look for the psychological status of the patients, however, although half of the respondents were optimistic about the support from their family and community but about one fourth felt socially neglected and 17.1% feels isolation within the family. 

## 5. Conclusion

Knowledge about cause and treatment of tuberculosis among TB patients was quite good, however, misconceptions also exist. Misconceptions about transmission of disease lead to discrimination like separate utensils for food or drink. Diagnosis of TB is associated with increase anxiety/tension, fear of loss of wage/earning, and stigma threatening self-esteem and quality of life. Mass media can be better utilized to remove misconceptions. Psychosocial reactions towards TB as revealed in this study should be addressed through counseling and communication during treatment in the DOTS centre. This may contribute to success of the national TB control program.

## Figures and Tables

**Table 1:** Sociodemographic characteristics and living condition.

	Male	Female
	(*n* = 485)	(*n* = 387)
Level of education		
Illiterate	25.8	24.5
Institutional	69.6	70.5
Informal	4.5	4.9
Family size	4.89 ± 1.8	5.03 ± 1.9
(Mean ± SD)	(1–13)	(1–12)
No. of living rooms in the house	1.72 ± 0.9	1.56 ± 0.8
(Mean ± SD)	(1–7)	(1–5)
Person living per room	3.27 ± 1.4	3.68 ± 1.6
(Mean ± SD)	(1–8)	(1–10)

**Table 2:** Distribution according to sex and sources of information about TB.

Sources of information	Male%	Female%	Total%
	(*n* = 485)	(*n* = 387)	(*n* = 872)
TV	46.4	47.3	46.8
Doctor's chamber	20.0	16.0	18.2
Family members/friends	14.2	15.0	14.6
Radio	13.4	13.7	13.5
Government Hospital	12.2	8.8	10.7
Billboard	10.1	7.0	8.7
NGO worker	8.5	7.2	7.9
Liflet, poster, and other printed materials	3.9	2.6	3.3
Pharmacy	.8	1.8	1.3

**Table 3:** Symptoms experienced during TB diagnosis.

Symptoms during TB Diagnosis	Male%	Female%	Total%
	(*n* = 485)	(*n* = 387)	(*n* = 872)
Night fever	91.5	87.9	89.9
Fatigue/tiredness	89.1	83.2	86.5
Productive cough	87.8	72.1	80.8
Loss of weight	70.5	68.2	69.5
Cough more than 3 weeks	66.6	55.3	61.6
Nausea	56.9	58.7	57.7
Severe headache	53.2	59.2	55.8
Chest pain	51.3	46.8	49.3
Shortness of breath	38.8	35.4	37.3
Fever without cause that lasts >7 days	34.2	30.0	32.3
Haemoptysis	27.8	22.5	25.5

**Table 4:** knowledge on ways of transmission of disease (TB).

Knowledge on transmission of disease	Male% (*n* = 485)	Female% (*n* = 387)	Total% (*n* = 872)
Do not know	18.4	28.2	22.9
Through sneezing/cough	57.1	54.5	56.0
Smoking	8.9	1.0	5.4
Dust	4.9	3.4	4.2
Unhygienic condition	2.9	3.4	3.1
Familial	1.9	2.6	2.2
Through cold	1.9	2.6	2.2
Handshake with TB patients	2.1	1.6	1.8
Irregular diet	.6	1.0	.9
Eating from the same plate	.2	1.0	.6

**Table 5:** Perception about how TB would be cured.

	Male%	Female%	Total%
	(*n* = 485)	(*n* = 387)	(*n* = 872)
TB could be cured completely	98.6	97.2	97.9
Remedial measure of TB			
Specific drugs given by health centre/DOTS	96.9	98.2	97.5
Do not know	2.1	1.0	1.6
Herbal remedies	.2	.3	.2
Praying	.8	.5	1.3

**Table 6:** Feeling about other TB patients.

Perception about the TB	Male%	Female%	Total%
	(*n* = 485)	(*n* = 387)	(*n* = 872)
I feel compassion and desire to help	66.6	64.6	65.7
I have no particular feeling	28.0	29.2	28.6
I tend to stay away from these people	4.1	5.9	4.9
I fear them because they may infect me	.8	.3	.6

**Table 7:** Perception on being a TB patient.

Perception as TB patients	Male%	Female%	Total%
	(*n* = 485)	(*n* = 387)	(*n* = 872)
Family members are cooperative towards me	96.9	93.5	95.4
Fear chance of reduction of family income	66.6	50.1	59.3
Increase sadness	57.9	41.1	50.5
Utensils are separated for me	47.6	45.2	46.6
Threat of loss of job/wages	48.0	29.5	39.8
Feels socially neglected/ low esteem	17.9	25.8	21.4
Most people behave differently	11.8	13.4	12.5
Feel isolated within the family	6.6	17.1	11.2
Family member avoid me	1.2	5.2	3.0

## References

[1] (2011). Global tuberculosis control: epidemiology, strategy, financing. *WHO Report*.

[2] Christopher O, Bosede I (2010). Health seeking behaviour of tuberculosis patients in Ekiti State, Nigeria. *Studies on Ethno-Medicine*.

[4] (1993). Resolution WHA44. 8: Tuberculosis control programme. *Handbook of Resolutions and Decisions of the World Health Assembly and the Executive Board*.

[5] Tuberculosis control in Bangladesh (2009). National tuberculosis control program. *Annual Report 2008*.

[6] Karim F, Johansson E, Diwan VK, Kulane A (2011). Community perceptions of tuberculosis: a qualitative exploration from a gender perspective. *Public Health*.

[7] Ganapathy S, Thomas BE, Jawahar MS, Selvi KJ, Sivasubramaniam, Weiss M (2008). Perceptions of gender and tuberculosis in a south Indian urban community. *The Indian Journal of Tuberculosis*.

[8] Ahsan G, Ahmed J, Singhasivanon P (2004). Gender difference in treatment seeking behaviours of tuberculosis cases in rural communities of Bangladesh. *Southeast Asian Journal of Tropical Medicine and Public Health*.

[9] Malhotra R, Taneja DK, Dhingra VD, Rajpal S, Mehra M (2002). Awareness regarding tuberculosis in a rural population in Delhi. *Indian Journal of Community Medicine*.

[10] Buregyeya E, Kulane A, Colebunders R (2011). Tuberculosis knowledge, attitudes and health-seeking behaviour in rural Uganda. *International Journal of Tuberculosis and Lung Disease*.

[11] Mushtaq MU, Shahid U, Abdullah HM (2011). Urban-rural inequities in knowledge, attitudes and practices regarding tuberculosis in two districts of Pakistan's Punjab province. *International Journal for Equity in Health*.

[12] Croft RP, Croft RA (1999). Knowledge, attitude and practice regarding leprosy and tuberculosis in Bangladesh. *Leprosy Review*.

[13] Mesfin MM, Tasew TW, Tareke IG, Mulugeta GWM, Richard JM (2005). Community knowledge, attitudes and practices on pulmonary tuberculosis and their choice of treatment supervisor in Tigray, Nothern Ethiopia. *The Ethiopian Journal of Health Development*.

[14] Edginton ME, Sekatane CS, Goldstein SJ (2002). Patients' beliefs: do they affect tuberculosis control? A study in a rural district of South Africa. *International Journal of Tuberculosis and Lung Disease*.

[15] Weiss MG, Auer C, Somma DB, Abouhia A (2006). *Gender and Tuberculosis: Cross-Site Analysis and Implications of a Multi-Country. Study in Bangladesh, India, Malawi and Colombia*.

[16] Kilale AM, Mushi AK, Lema LA (2008). Perceptions of tuberculosis and treatment seeking behaviour in Ilala and Kinondoni Municipalities in Tanzania. *Tanzania Journal of Health Research*.

[17] Long NH, Johansson E, Diwan VK, Winkvist A (1999). Different tuberculosis in men and women: beliefs from focus groups in Vietnam. *Social Science and Medicine*.

[18] Shetty N, Shemko M, Abbas A (2004). Knowledge, attitudes and practices regarding tuberculosis among immigrants of Somalian ethnic origin in London: a cross-sectional study. *Communicable Disease and Public Health*.

[19] Manoharan E, John KR, Joseph A, Jacob KS (2001). Psychiatric morbidity, Patients perspectives of illness and factors associated with poor medication compliance among the tuberculosis in Vellore, South India. *The Indian Journal of Tuberculosis*.

[20] Eram U, Khan IA, Md Tamanna Z, Khan Z, Khaliq N, Abidi AJ (2006). Patient perception of illness and initial reaction to the diagnosis of tuberculosisIndian. *Journal of Community Medicine*.

[21] Macfarlane L, Newell NJ (2012). A qualitative study exploring delayed diagnosis and stigmatization of tuberculosis amongst women in Uganda. *Intenational Health*.

